# Monofunctional and Interstrand DNA Adducts of Platinum(II) Complexes

**DOI:** 10.1155/MBD.1994.195

**Published:** 1994

**Authors:** Viktor Brabec, Vladimír Boudny

**Affiliations:** 1 Institute of Biophysics Academy of Sciences of the Czech Republic Kralovopolska 135 Brno 612 65 Czech Republic

## Abstract

The effects produced in DNA by monofunctional or interstrand adducts of platinum(II) complexes have been summarized. The monofunctional adducts destabilize DNA in a sequence-dependent manner via conformational distortions, which may have a denaturational character. It has been suggested that this conformational alteration facilitates in DNA the formation of the bidentate DNA adducts, whose formation is associated with a permanent or transient local denaturation. In addition, DNA interstrand cross-linking by *cis*-diamminedichloroplatinum(II) and its *trans* isomer produce in DNA lesions that have quite different characteristics. It has been suggested that these differences may have relevance to the distinct antitumor efficacy of the two platinum isomers.


### MONOFUNCTIONAL AND INTERSTRAND DNA ADDUCTS OF PLATINUM(II) COMPLEXES

Viktor Brabec* and Vladimír Boudny

Institute of Biophysics, Academy of Sciences of the Czech Republic, Kralovopolska 135, 612 65 Brno, Czech Republic

**ABSTRACT.** The effects produced in DNA by monofunctional or interstrand adducts of platinum(II) complexes have been summarized. The monofunctional adducts destabilize DNA in a sequence-dependent manner via conformational distortions, which may have a denaturational character. It has been suggested that this conformational alteration facilitates in DNA the formation of the bidentate DNA adducts, whose formation is associated with a permanent or transient local denaturation. In addition, DNA interstrand cross-linking by *cis*-diamminedichloroplatinum(II) and its *trans* isomer produce in DNA lesions that have quite different characteristics. It has been suggested that these differences may have relevance to the distinct antitumor efficacy of the two platinum isomers.

### INTRODUCTION

Platinum cytostatics are widely used for cancer chemotherapy alone and in combination with other drugs (1). Numerous studies suggest that the cytotoxic action of platinum drugs is related to its ability to react with cellular DNA (2-5). The platinum drugs bind to DNA preferentially to guanine residues at the N(7) position, producing monofunctional adducts that can subsequently close to bifunctional lesions (6-8). Most of the structural information currently available pertains to the intrastrand cross-links of platinum between adjacent purines. 90 % of these adducts are found in the reaction of linear DNA with *cis*-diamminedichloroplatinum(II) (8,9) {cisplatin, *cis*-$[Pt(NH_3)_2Cl_2]$}, which is one of the most effective anticancer drugs. Therefore, these intrastrand lesions are believed to represent major DNA adducts of platinum drugs. Monofunctional lesions, interstrand cross-links and intrastrand cross-links between two nonadjacent purines are considered the minor DNA adducts of clinically effective platinum drugs.

Clinically ineffective *trans* isomer of cisplatin {*trans*-[Pt($NH_3$)$_2$$Cl_2$]} does not form intrastrand cross-links between adjacent purines for sterical reasons (10). It has been, therefore, speculated that the latter DNA adducts of clinically effective cisplatin and its simple analogues are most likely responsible for antitumor activity of these drugs. Nevertheless, the DNA lesion (or lesions) of cisplatin and its simple analogues still remains (or remain) to be established conclusively.

Chlorodiethylenetriamineplatinum(II) chloride (dien-Pt) is the monodentate complex, used to afford DNA monofunctional adducts to simulate the first step of the bifunctional reaction of *cis*- or *trans*-[Pt($NH_3$)$_2$$Cl_2$] and their simple analogues with DNA. It has been almost axiomatic that the monofunctional DNA adducts of platinum complexes do not affect DNA conformation. This view has been related to antitumor inefficiency of monofunctional platinum complexes and persists in spite of some results indicating that the monofunctional platinum(II) adducts change the conformation of DNA.

The bifunctional platinum complexes can form DNA interstrand cross-links. Interestingly, the frequency of interstrand cross-links of cisplatin has been correlated with its cytotoxicity in several biological systems (11,12). In spite of these results the interstrand cross-links of platinum complexes are not considered the lesions responsible for antitumor effect. This view is mainly derived from the observation that clinically ineffective *trans*-[Pt($NH_3$)$_2$$Cl_2$] forms more interstrand cross-links than does cisplatin (13). The latter speculation is, however, based on the assumption that both isomers form identical interstrand DNA lesions. However, DNA interstrand cross-links of platinum complexes have been studied less thoroughly so that the latter assumtion has no experimental support.

In the present paper, we summarize our recent findings describing (i) distortions produced in DNA by the monofunctional adducts of platinum complexes and (ii) characteristics of interstrand cross-links of *trans*-[Pt($NH_3$)$_2$$Cl_2$].

### RESULTS

Sequence-Dependent Distortions Induced in DNA by Monofunctional Platinum(II) Binding (14,15).

The effects on thermal stability and conformation of DNA produced by the monofunctional adducts of dien-Pt have been investigated. Oligodeoxyribonucleotide duplexes of varying lengths [9 - 20 base pairs (bp)] and of varying central trinucleotide sequences were prepared and characterized that contained site-specific and unique N7-guanine adduct. Included are adducts at the sequences of d(AGC), d(AGT), d(CGA), d(CGT), d(TGA), d(TGC) and d(TGT). All these monofunctional adducts decrease the melting temperature ($T_m$) of the duplexes. This destabilization effect exhibits a sequence-dependent variability. The highest lowering of $T_m$ is observed for the modified duplexes containing the central sequence of Py-deoxyriboguanosine-Py (Py is a pyrimidine deoxyribonucleoside). The destabilization effect is reduced with decreasing concentration of $Na^+$. Polarography, circular dichroism, phenantroline-copper and chemical probes reveal conformational distortions spreading over several base pairs around the adduct. The effects of monofunctional platinum(II) adducts on conformational distortions in DNA exhibit a similar sequence-dependent variability as on thermal stability of DNA. The influence of the monofunctional adduct formed by *cis*-[Pt$(NH_3)_2Cl_2$] on the stability of the oligonucleotide duplex has been also studied. This lesion decreases thermal stability of DNA in the same way as the adduct of dien-Pt.

The effects on conformation of DNA produced by the monofunctional adducts of chlorodiethylenetriamine-platinum(II) chloride or *cis*-diamminemonoaquamonochloro-platinum(II) have been also investigated by means of the single-strand-specific probe, chloroacetaldehyde (CAA). The denatured sites to which CAA was bound and that were induced in DNA by the monofunctional adducts of the platinum complexes, were characterized by means of the three experimental approaches. These include measurement of fluorescence of the plasmid fragment treated with CAA, analysis of oligonucleotides treated with CAA and cleaved by piperidine and termination of duplex transcription on fragment of plasmid DNA treated with CAA. The results indicate that the denaturational change preferentially occurs in the base pair containing the monoadducted deoxyriboguanosine in the trinucleotide sequence Py-deoxyriboguanosine-Py. It was suggested that this conformational alteration facilitates in DNA the formation of minor bifunctional adducts of *cis*-diamminedichloroplatinum(II).

<u>DNA interstrand cross-links of *trans*-diamminedichloro-platinum(II) are preferentially formed between guanine and complementary cytosine residues</u> (16).

Bases in the opposite strands of DNA cross-linked by clinically ineffective *trans*-[Pt$(NH_3)_2Cl_2$]} have been identified by means of three experimental approaches. These include HPLC analysis of enzymatic digests of synthetic oligonucleotide duplexes containing the interstrand cross-link, footprinting experiments on the interstrand cross-linked oligonucleotide duplexes and termination of the duplex transcription on *trans*-[Pt$(NH_3)_2Cl_2$]-treated fragments of plasmid DNA. The results reveal that deoxyriboguanosine and complementary deoxyribocytidine residues are preferential binding sites of *trans*-[Pt($NH_3$)$_2$$Cl_2$] in the interstrand adducts. The kinetics of the interstrand cross-linking reaction was studied by means of gel electrophoresis for the *cis* and *trans* isomers. The initial rate was markedly lower in the case of the *trans* isomer {$t_{1/2}$ = c. 11 hr in comparison with 4 hr found for *cis*-[Pt($NH_3$)$_2$$Cl_2$]}. On the other hand, after 48 hr *trans*-[Pt($NH_3$)$_2$$Cl_2$] forms about twice amount of interstrand cross-links in linear DNA as compared with the *cis* isomer after 48 hr.

<u>DNA Conformational Change Produced by Site-Specific Interstrand Cross-Link of *trans*-Diamminedichloroplatinum(II)</u> (17).

The DNA distortion produced by the interstrand cross-link of *trans*-[Pt($NH_3$)$_2$$Cl_2$] has been described by means of gel electrophoresis, chemical probes and molecular mechanics modeling. Synthetic double-stranded oligodeoxyribonucleotides of varying lengths (19 - 22 bp) were synthesized that contained a unique site-specific interstrand cross-link within their central sequence d(TGCT)/d(AGCT) between complementary guanine and cytosine residues. We find that the platinated deoxyriboguanosine residue adopts *syn* conformation. The duplex is distorted on both sides of the cross-link but the bases are still paired. The distortion introduces some flexibility into the helix. In addition, the double helix is unwound by approximately $12^{o}$ and bent towards the major groove by $26^{o}$.

### DISCUSSION

The results summarized in this paper indicate that the monofunctional platinum(II) binding induces extensive conformational distortions in double-helical DNA, which are, however, qualitatively different from those induced by bifunctional platinum(II) intrastrand cross-links between adjacent purine residues. These effects on DNA may have biological relevance. The latter conclusion is supported by the observation that monofunctional DNA adducts of platinum are recognized and repaired in assays with (A)BC excinuklease even with 2-3 times greater efficiency than the bifunctional and major adducts of cis-[Pt($NH_3$)$_2$$Cl_2$] (18).

This paper also summarizes the results of our recent studies aimed at describing the properties of interstrand cross-links of *trans*-[Pt($NH_3$)$_2$$Cl_2$] (Table I). Table I also compares the characteristics of these lesions with those produced by the interstrand cross-links of *cis*-[Pt($NH_3$)$_2$$Cl_2$] described more recently (18,19).

*cis*- and *trans*-[Pt($NH_3$)$_2$$Cl_2$] behave quite differently with respect to the properties of DNA interstrand cross-links. These results may have relevance to the clinical activity of the platinum drugs. This view is also supported by our preliminary results demonstrating that *cis*-$[Pt(NH_3)_2Cl_2]$ forms preferentially interstrand, not intrastrand cross-links in supercoiled DNA at low levels of its modification.

Table I: Properties of the interstrand cross-links formed in DNA by *cis*- or *trans*-$[Pt(NH_3)_2Cl_2]$

| | *cis*-$[Pt(NH_3)_2Cl_2]$ | *trans*-$[Pt(NH_3)_2Cl_2]$ | Reference |
|---|---|---|---|
| Sites in the cross-link: | 5'-**G**C-3' \ 3'-C**G**-5' | -**G**- \| -**C**- | 16, 19 |
| Bending: | c. 55° towards major groove | 26° towards major groove | 17, 20 |
| Unwinding: | 0° | 12° | 17, 20 |
| Distortion: | Localized at the platinated sequence d(GC)/d(GC) | Nondenaturational, extending over c. 4 bp around the cross-link, platinated dGuo in syn conformation, limited flexibility of the duplex | 17, 20 |
| Quantity*): | c. 6 % | c. 12 % | 16 |
| Kinetics*): | $t_{1/2}$ = 4 hr | $t_{1/2}$ > 11 hr | 16 |

*) In linearized plasmid DNA (2455 bp) at $r_b$ = 0.001

**REFERENCES**

1. Loehrer, D.M.J., & Einhorn, L.H. (1984) Ann. Intern. Med. **100**, 704-713.
2. Eastman, A. (1987) (1987) Pharmacol. Ther. **34**, 155-166.
3. Johnson, N.P., Butour, J.L., Villani, G., Wimmer, F.L., Defais, M., Pierson, V., & Brabec, V. (1989) in *Progress in Clinical Biochemistry and Medecine*, Vol. 10, pp 1-24, Springer, Berlin.
4. Lepre, C.A., & Lippard, S.J. (1990) in *Nucleic Acids and Molecular Biology* (Eckstein, F., & Lilley, D.M.J., Eds) Vol. 4, pp 3-9, Springer, Berlin.
5. Reedijk, J. (1987) Pure Appl. Chem. **59**, 181-192.
6. Bancroft, P.D., Lepre, C.A., & Lippard, S.J. (1990), J. Am. Chem. Soc. **112**, 6860-6871.

7. Brabec, V., Kleinwachter, V., Butour, J.L., & Johnson, N.P. (1990) Biophys. Chem. **35**, 129-141.
8. Eastman, A. (1986) Biochemistry **25**, 3912-3915.
9. Fichtinger-Schepman, A.M.J., van der Veer, J.L., den Hartog, J.H.J., Lohman, P.H.M., & Reedijk, J. (1986) Biochemistry **28**, 7975-7989.
10. Eastman, A., & Jennerwein, M.M. (1988) Chem.-Biol. Interact. **67**, 71-80.
11. Roberts, J.J. (1987) in *Molecular Mechanisms of Carcinogenic and Antitumor Activity* (Chagas, C., & Pullman, B., Eds) pp 463-489, Pontifica Academia Scientarum, Vatican City.
12. Roberts, J.J., Knox, R.J., Pera M.F., Friedlos, F., & Lydall, P.A. (1988) in *Platinum and Other Metal Coordination Compounds in Cancer Chemotherapy* (Nicolini, M., Ed) pp 16-31, Nijhoff, Boston.
13. Eastman, A. (1982) Biochem. Biophys. Res. Commun. **105**, 869-875.
14. Brabec, V., Reedijk, J., & Leng, M. (1992) Biochemistry **31**, 12397-12402.
15. Brabec, V., Boudny, V., & Balcarova, Z. (1993) Biochemistry, submitted.
16. Brabec, V., & Leng, M. (1993) Proc. Natl. Acad. Sci. USA **90**, 5345-5349.
17. Brabec, V., Sip, M., & Leng., M. (1993) Biochemistry, in press.
18. Page, J.D., Husain, I., Sancar, A., & Chaney, S.G. (1990) Biochemistry **29**, 1016-1024.
19. Lemaire, M.A., Schwartz, A., Rahmouni, A. R., & Leng, M. (1991) Proc. Natl. Acad. Sci. USA **88**, 1982-1985.
20. Sip., M., Schwartz, A., Vovelle, F., Ptak, M., & Leng, M. (1992) Biochemistry **31**, 2508-2513.

**Received: September 20, 1993**

